# Surgical treatment for pulmonary metastasis from ovarian cancer: a retrospective case series

**DOI:** 10.1186/s40792-024-01927-5

**Published:** 2024-05-27

**Authors:** Saki Tsubouchi, Yo Tsukamoto, Ai Ishikawa, Rintaro Shigemori, Daiki Kato, Takamasa Shibazaki, Shohei Mori, Takeo Nakada, Makoto Odaka, Takashi Ohtsuka

**Affiliations:** 1 Department of Surgery, The Jikei University School of Medicine, Nishishinbashi 3-19-18, Minatoku, Tokyo 105-8471 Japan; 2 Department of Surgery, The Jikei University Kashiwa Hospital, 163-1 Kashiwashita Kashiwashi, Chiba, 277-8567 Japan

**Keywords:** Pulmonary metastasis, Ovarian cancer, Carbohydrate antigen 125

## Abstract

**Background:**

Distant metastases of ovarian cancer are rarely detected alone. The effectiveness of surgical intervention for pulmonary metastases from ovarian cancer remains uncertain. This study aimed to investigate the clinicopathologic characteristics and outcomes of patients undergoing resection for pulmonary metastasis from ovarian cancer.

**Case presentation:**

The clinicopathologic characteristics and outcomes of radical surgery for pulmonary metastasis from ovarian cancer were investigated. Out of 537 patients who underwent pulmonary metastasis resection at two affiliated hospitals between 2010 and 2021, four (0.74%) patients who underwent radical surgery for pulmonary metastasis from ovarian cancer were included. The patients were aged 67, 47, 21, and 59 years; the intervals from primary surgery to detection of pulmonary metastasis from ovarian cancer were 94, 21, 36, and 50 months; and the overall survival times after pulmonary metastasectomy were 53, 50, 94, and 34 months, respectively. Three of the four patients experienced recurrence after pulmonary metastasectomy. Further, preoperative carbohydrate antigen (CA) 125 levels were normal in two surviving patients and elevated in the two deceased patients.

**Conclusion:**

In this study, three of the four patients experienced recurrence after pulmonary metastasectomy, but all patients survived for > 30 months after surgery. Patients with ovarian cancer and elevated CA125 levels may not be optimal candidates for pulmonary metastasectomy. To establish appropriate criteria for pulmonary metastasectomy in patients with ovarian cancer, further research on a larger patient cohort is warranted.

## Introduction

Ovarian cancer (OC) has a poor prognosis, especially as it is often diagnosed at an advanced stage in more than 70% of cases, and at least 60% of patients experience recurrence after the primary treatment [1]. Most cases of ovarian cancer recur intraperitoneally, and distant metastases are rarely isolated [1, 2]. Therefore, there are only a few reports on the resection of pulmonary metastases (PMs) from OC, including a few case reports and small cohort studies. There is a paucity of reports on pulmonary metastasis (PM) resection from OC scheduled for radical resection. The effectiveness of surgical intervention for PMs from OC is unclear. This study aimed to investigate the clinicopathological features and prognoses of patients undergoing resection for PM from OC.

## Patients and methods

This retrospective study investigated the clinicopathologic findings and prognosis of OC after the radical resection of PMs. We reviewed patients who underwent surgery for PMs at two affiliated hospitals, The Jikei University Hospital and The Jikei University Kashiwa Hospital, between January 2010 and December 2021. Patients who underwent interventions only for the purpose of biopsy were excluded. Surgery was indicated per Thomford's criteria [3] as follows: (1) good general health for lung surgery; (2) controlled primary tumor; (3) absence of metastases in organs other than the lungs, or if present, it should be controlled through surgery or other treatments; and (4) completely removable PMs. At our institution, surgery for PMs was considered if there were no extra-abdominal metastases other than the pulmonary lesion. Patients with intraperitoneal metastases before pulmonary metastasectomy (PMT) were considered to be under control if the intraperitoneal metastases were reduced, disappeared, or remained stable on follow-up computed tomography (CT) scans after treatment.

This study was approved by the institutional review board of the Jikei University School of Medicine [approval number: 31-384(9964)] on November 4, 2022. Informed consent was waived because of the retrospective nature of the study. The overall survival (OS) starting date was the date of PMT, the event was the date of death, and the cutoff date was the date of the patient’s last visit to the hospital.

Surveillance after OC treatment was performed according to the Japanese Society of Gynecologic Oncology guidelines, which complies with the National Comprehensive Cancer Network guidelines [4]. After initial OC treatment, patients were followed up every 2–4 months for the first 2 years, every 3–6 months for the next 3 years, and every year after the 5th year. Follow-up examination included internal examination and blood tests as well as imaging assessment when clinically indicated.

The date of PM detection was the date when a lesion suspected of being a PM was first noted on CT scan, which was determined by an experienced thoracic surgeon or radiologist. Each patient’s age, primary pathology, primary OC stage, presence or absence of chemotherapy for OC, presence or absence of recurrent metastasis to other organs before PM resection, pattern of PM (number, location and left or right), surgical procedure and perioperative complications of PM resection, presence or absence of recurrent metastasis after resection of PM, treatment for recurrence, pre-PM resection tumor marker (carcinoembryonic antigen (CEA), carbohydrate antigen (CA)19-9, carbohydrate antigen (CA) 125) levels, and cause of death were recorded.

## Results

Between January 2010 and December 2021, 537 patients underwent resection for PMs at two affiliated institutions, including five of them who underwent surgery for PM from OC. One of these patients who had undergone surgery for the purpose of biopsy was excluded; the remaining four were reviewed. The characteristics of our study participants are summarized in Table 1.

**Table 1:** Characteristics of participants

Case	Age	Histology	Primary stage	Adjuvant chemotherapy after primary surgery	Interval from initial surgery for OC to the detection of PM (month)	Recurrence before PMT	CEA before PMT (ng/mL)	CA19-9 before PMT (U/mL)	CA125 before PMT (U/mL)
1	67	Adenocarcinoma	III	Yes	94	No	5.1	14	311
2	47	Clear cell carcinoma	II	Yes	21	No	1.9	31	3
3	21	Mucinous borderline tumor	I	No	42	Peritoneal dissemination	2	1	5
4	59	Serous cystadenocarcinoma	I	No	50	Intraperitoneal lymph node	1.1	14	161

Preoperative diagnosis	Surgical procedure for PMT	Number of PM	Pathological size of PM (mm)	Additional treatment after PMT	Recurrence after PMT	Treatment for recurrence after PMT	DFI after PMT (month)	OS after PMT (month)	Outcome
Metastasis	Lobectomy	1	35	Chemotherapy	Peritoneal dissemination	Chemotherapy	13	53	Dead
Metastasis	Wedge resection	1	21	Chemotherapy	Mediastinal lymph nodes	Surgery	48	50	Survival
Primary or metastasis	Lobectomy	1	12	Chemotherapy	No	No	94	94	Survival
Metastasis	Wedge resection	1	15	Chemotherapy	Pelvic lymph node	Surgery	4	34	Dead

OC: ovarian cancer; DFI: disease-free interval; PMT: pulmonary metastasectomy; CEA: carcinoembryonic antigen; CA19-9: carbohydrate antigen 19-9; CA125: cancer antigen 125; PM: pulmonary metastasis; DFS: disease-free interval; OS: overall survival

## Case presentation

### Patient 1

The patient was 67 years old at the time of PMT. The primary ovarian cancer was adenocarcinoma, and the pathological stage was stage III when adjuvant chemotherapy was administered. Ninety-four months after OC surgery, chest CT scan revealed one nodule with the first suspected metastasis. CA125 levels were elevated (311 U/mL) before PMT. We performed lobectomy because the lesion was located in the hilar region of the left upper lobe. Postoperative pathology revealed a lesion measuring 35 mm in diameter, and a diagnosis of PM from the OC was made. Pathological examination revealed no metastases in the hilar or mediastinal lymph nodes. Chemotherapy was administered after PMT. Thirteen months after PMT, peritoneal dissemination was observed, and the patient received chemotherapy, but died of ovarian cancer 53 months after PMT.

### Patient 2

The patient was 47 years old at the time of PMT. The primary ovarian cancer was a clear cell carcinoma, and the pathological stage was Stage II when adjuvant chemotherapy was administered. Twenty-one months after the ovarian cancer surgery was performed, a chest CT scan revealed one nodule with the first suspected metastasis. Tumor marker levels were not elevated before PMT. We performed wedge resection because the region of metastasis was in the peripheral part of the lung. Postoperative pathology revealed a lesion measuring 21 mm in diameter, and a diagnosis of PM from the OC was made. Chemotherapy was administered after PMT. Forty-eight months after PMT, mediastinal lymph node metastasis was found, for which we performed a lymphadenectomy. The patient is alive 50 months after PMT.

### Patient 3

The patient was 21 years old at the time of PMT. The primary ovarian cancer was a mucinous borderline tumor with pathological stage I. Adjuvant chemotherapy was not administered. Peritoneal dissemination was found six months after the initial OC surgery, for which chemotherapy and interval debulking surgery were performed. Thereafter, no recurrence was observed. Forty-two months after the initial surgery, one nodule with suspected malignancy was found in the lung. Intraperitoneal metastases disappeared on CT before the PMT. Tumor marker levels were not elevated before PMT. When making a preoperative diagnosis, it was difficult to determine whether the lesion was a PM from OC or primary lung cancer. Intraoperative frozen section analysis was performed, but the diagnosis could not be confirmed; hence, lobectomy and mediastinal lymph node dissection were performed. Postoperative pathology revealed a lesion measuring 12 mm in diameter, and the diagnosis of PM from OC with no metastases in the hilar and mediastinal lymph nodes was made. Chemotherapy was administrated after PMT. The patient was alive 94 months after the PMT, with no recurrence.

### Patient 4

The patient was 59 years old at the time of PMT. The primary ovarian cancer was a serous cystadenocarcinoma, and the pathological stage was stage I; therefore, adjuvant chemotherapy was not administered. Intraperitoneal lymph node metastases and PM were found on CT scans 50 months after the initial OC surgery, and chemotherapy was administered. The pelvic lymph nodes were reduced on CT, but the PM was found to be enlarged; therefore, we decided to perform PMT. CA125 levels were elevated (161 U/mL) before PMT. We performed wedge resection because the metastatic region was in the peripheral part of the lung. Postoperative pathology revealed a lesion measuring 15 mm in diameter, and the patient was diagnosed with PM originating from the OC. Chemotherapy was administrated after PMT. Four months after the PMT, the patient was found to have re-growing pelvic lymph node metastases and underwent lymphadenectomy. Chemotherapy was administered, but the patient died of ovarian cancer 34 months after PMT regardless.

No perioperative complications were observed in any of the patients. Three of the four patients presented with recurrence after PMT, but still achieved an approximate 3-year survival rate. Patients 1 and 4 died from OC 53 and 34 months after PMT. Preoperative CA125 levels were not elevated in the two surviving patients, but were elevated in the two deceased patients, whereas CEA and CA19-9 levels were not elevated in any of the patients.

## Discussion

We reviewed the prognoses of patients who underwent radical surgery for PM from OC at the two affiliated centers. Three of 4 included patients presented with recurrence after PMT, but all patients had a survived time of > 30 months after PMT. Of the four patients who underwent resection of PM, two with elevated CA125 levels before PMT died after surgery. This suggests that pre-PMT CA125 levels could be a prognostic factor after surgery for PM from OC.

OC has one of the poorest prognoses among all gynecological malignancies. Up to 65% of patients with OC are diagnosed at an advanced stage, and for them, the cure rate is 18% (the SEER database) [5]. Eighty-five percent of patients with OC recurrence present with intra-abdominal disease, resulting in death due to ascites or bowel obstruction [6]. Because PMs from OC are usually accompanied by extra-thoracic metastases and solitary PMs are rare, there have been only a few studies on radical surgery for PM from OC [7]. According to the 2016 Japanese statistics, only 69 (0.8%) of 8497 patients underwent PMT for OC [8]. In our study, two patients had isolated PMs and two others had PMs followed by intra-abdominal metastasis. In the latter cases, PMT was performed because intra-abdominal metastases were controlled through interval debulking surgery and chemotherapy. Three of the four patients in this study experienced post-PMT recurrence but survived for three years. Tumor-reducing surgery and chemotherapy have been shown to prolong overall survival (OS) in patients with recurrent OC compared with chemotherapy alone [9]. Similarly, PMT followed by chemotherapy may contribute to improved OS. Notably, PMT alone is not a comprehensive management strategy for PMs in OC patients. As chemotherapy is a crucial treatment option for OC recurrence, the indication for surgery for PMs and the surgical procedure should be carefully determined. In this study, although all patients received chemotherapy after PMT, only one of them achieved an OS of 94 months. The results of previously reported cases of PMs caused by OC are summarized in Table 2. To the best of our knowledge, there are only a few reports of PMT only in OC, [6, 10–13] but all indicate a good prognosis after PMT, with some reporting long-term OS exceeding 5 years.

**Table 2:** Reported cases of pulmonary metastasectomy for ovarian cancer

Reporter	Reporting year	Number of cases of PMT from OC	Median DFI after primary surgery (month)	Median DFI after PMT (month)	Median OS after PMT (month)
1. Kimura 8)	1995	1	75	14	23
2. Toishi 9)	2011	1	154	11	15
3. Kita 10)	2015	1	84	12	24
4. Adachi 11)	2015	5	28.5 (gynecological cancers)	18.8 (gynecological cancers)	5-year OS: 100%
5. Kanzaki 6)	2020	6	22	15	44
This study	2024	4	43	31	51

OC: ovarian cancer; DFI: disease-free interval; PMT: pulmonary metastasectomy; OS: overall survival

The interval from resection for primary cancer to the detection of PMs is an important prognostic factor after the resection of PMs from various cancer types [12]. Similarly, a long interval from primary OC resection to the detection of PMs has been suggested as a favorable prognostic factor for resection of PMs from OC [11, 13, 14]. This study differed from previous reports in terms of the interval from primary surgery to the detection of PMs, with a longer interval observed in the deceased patients and a shorter interval in the survivors. Because of the small number of patients in this study, no statistical analysis of the interval after primary cancer resection and OS could be conducted.

In this study, preoperative CA125 levels were elevated in the two deceased patients but not in the surviving patients, suggesting that elevated preoperative CA125 levels in OC is a prognostic factor. Serum CA125 levels are elevated in 50% of patients with early-stage OC and 92% of those with advanced-stage OC [15, 16]. Therefore, CA125 levels are commonly examined during the follow-up of patients with OC [17]. CA125 is soluble in serum as well as on the tumor cell surface. It also has a half-life of approximately two weeks, which allows for a precise examination [18]. This makes it useful in OC diagnosis, treatment efficacy evaluation, and monitoring of pathological conditions. In patients with elevated CA125 levels, subtle (cellular) systemic metastases may be present in addition to resectable PMs, even if imaging studies indicate that the malignancy is under control. When CA125 levels are elevated before primary surgery for OC and a solitary PM is present, a certain observation period should be considered to confirm whether the metastasis is indeed solitary. In addition to PM resection, postoperative adjuvant chemotherapy should be considered.

A multicenter trial is warranted for further investigation as the incidence of PMs from OC is rare at a single institution. Furthermore, future prospective studies should be conducted to explore the role of adjuvant chemotherapy after surgery for PMs.

This study has several limitations. The rarity of PMs from OC caused a significant selection bias in choosing patients for surgery for PMs from OC. Therefore, cases of PMs from OC for which surgery is indicated are scarce, making it difficult to accumulate cases at a single center or a few centers; hence, further multicenter studies are needed to validate the findings of this study.

## Conclusion

In this study, three of four patients experienced a recurrence after PMT, but all patients survived for > 30 months after PMT. Patients with high CA125 levels before resection of PM from OC may not be optimal candidates for PMT. To establish appropriate criteria for PMT in OC, further research with a larger patient cohort is necessary.

## Data Availability

The authors declare that all the data are described in this article and available upon reasonable request.

## Abbreviations

CA 125: Carbohydrate antigen 125
CA 19-9: Carbohydrate antigen 19-9
CEA: Carcinoembryonic antigen
OC: Ovarian cancer
OS: Overall survival
PM: Pulmonary metastasis
PMs: Pulmonary metastases
PMT: Pulmonary metastasectomy
